# Supplementary material for: The Terminal Extensions of Dbp7 Influence Growth and 60S Ribosomal Subunit Biogenesis in Saccharomyces cerevisiae
Source: Int J Mol Sci. 2023 Feb 9;24(4):3460. doi: 10.3390/ijms24043460 (PMC9960301; doi:10.3390/ijms24043460)
Supplement: Supplementary file 1 [file ijms-24-03460-s001.zip › ijms-2201922-supplementary.pdf]

Supplementary Tables and Figures

Table S1. Yeast strains used in this work.

Table S2. Plasmids used in this work.

Figure S1. Prediction of intrinsically disordered regions in Dbp7. (**A**) Prediction of the presence of disordered regions. Horizontal lines indicate disorder thresholds in each prediction engine. Red and blue dots indicate residues above and below the disorder threshold, respectively. Predictions using PrDOS (top panel) [5] and DisEMBL 1.5 (second, third and bottom panels showing respectively disorder predictions using the loops/coils, hot-loops or Remark-465 definitions, respectively) [6] are shown. (**B**) Structure prediction of Dbp7 using the PDB file provided for the Dbp7 protein of *Saccharomyces cerevisiae* (strain S288c) by AlphaFold Protein Structure Database [7,8]. The cartoon was generated with the UCSF Chimera program [9]. The RecA-1 and RecA-2 domains from the helicase core are coloured in blue and gold, respectively. Different motifs are indicated and highlighted in red, blue and green. The N-terminal extension is coloured in black, except the NLS that is coloured in pale blue. The C-terminal extension is coloured in green, except the DUF4217 domain that is coloured in yellow.

Figure S2. Cartoon representation of the structure of the different truncated versions of Dbp7 proteins of this study. Note that the implicated residues were hidden from the predicted structure of the full-length wild-type Dbp7 protein deposited in AlphaFold Protein Structure Database. No simulation of how the truncations modified the structure of the remaining protein sequence was undertaken.

Figure S3. Steady-state levels of the different truncated Dbp7 proteins. Strain JuCY1 transformed with different plasmid-borne *DBP7* alleles: HA-*DBP7* (wild-type control), HA-*dbp7ΔNLS,* HA-*dbp7ΔN10*, HA-*dbp7ΔN162*, and HA-*dbp7ΔC636-742* was grown in liquid SD-Trp medium at 30 °C and harvested at an OD_{600} of 0.8; whole cell extracts were prepared and equivalent amounts of protein from the different cell extracts were subjected to western blotting analyses with antibodies against the HA epitope. Pgk1 and Nhp2 were detected using specific antibodies and used as loading controls.

Figure S4. Immundetection of GFP-fused N-terminal Dbp7 constructs and the respective positive and negative controls. Whole cell extracts were prepared from YKL500 cells transformed with the indicated constructs: None (untransformed cells), empty pADH111-$(GA)_5$-3xyEGFP plasmid (Vector), pADH111-derived plasmid containing the NLS of the SV40 large T-antigen fused to 3xyEGFP (SV40-NLS), pADH111-derived plasmid containing the N-terminal domain of Dbp7 (from M1 to M162) fused to 3xyEGFP (Dbp7.N162) and pADH111-derived plasmid containing the N-terminal domain of Dbp7 (from M1 to M162) but lacking the segment from V48 to S78 (Dbp7.N162(ΔNLS)). Transformants were grown to exponential phase in liquid SD-Leu medium at 30 °C and whole cell extracts were prepared. Equal amounts of extracts were resolved by SDS-PAGE and analysed by western blotting using a specific anti-GFP antibody. Pgk1, which was revealed with a monoclonal anti-Pgk1 antibody, was used as a loading control.

Figure S5. Detection of the HA-Dbp7ΔNLS protein variant lacking the V48 to S78 sequence. Whole cell extracts were prepared from the indicated strains, which were grown to exponential phase in liquid SD-Trp medium at 30 °C. Equivalent amounts of extracts were analysed by western blotting using a specific anti-HA antibody. As a loading control, Pgk1, which was revealed with a monoclonal anti-Pgk1 antibody, was used.

Figure S6. Detection of the different GFP-tagged Dbp7 variant proteins. Whole cell extracts were prepared from the YKL500 strain expressing the indicated, plasmid-borne GFP-tagged Dbp7 constructs under control of the cognate *DBP7* promoter. In addition, YKL500 cells were also transformed with the pADH111-$(GA)_5$-3xyGFP vector, which expresses a triple GFP from the strong *ADH1* promoter. Cells were grown to exponential phase in liquid SD-Leu medium at 30 °C. Equivalent amounts of extracts were analysed by western blotting using a specific anti-GFP antibody. Pgk1, which was revealed with a monoclonal anti-Pgk1 antibody, was used as a loading control. Note the difference of the expression of the $(GA)_5$-3xyEGFP reporter from either the *ADH1* or the *DBP7* promoter. The GFP blot was overexposed (ca. 100-fold) to visualize the levels of the GFP-tagged Dbp7 variant proteins.

Figure S7. The C-terminal truncations of Dbp7 are not dominant negative over wild-type Dbp7. Growth test of a wild-type W303-1B strain transformed with an empty YCplac22 vector (Vector) or different plasmids expressing the following *DBP7* alleles: HA-*DBP7* (wild-type control), HA-*dbp7ΔC694-742*, HA-*dbp7ΔC636-742*, HA-*dbp7ΔN10*, and HA-*dbp7ΔN162*. Strains were serially diluted fivefold and spotted on SD-Trp plates, which were incubated at 30 °C for 2.5 days.

Supplementary references

1. Thomas, B.J.; Rothstein, R. Elevated recombination rates in transcriptionally active DNA. *Cell* **1989**, *56*, 619-630.
2. Pillet, B.; García-Gómez, J.J.; Pausch, P.; Falquet, L.; Bange, G.; de la Cruz, J.; Kressler, D. The dedicated chaperone Acl4 escorts ribosomal protein Rpl4 to its nuclear pre-60S assembly site. *PLoS Genet.* **2015**, *11*, e1005565.
3. Gietz, R.D.; Sugino, A. New yeast-*Escherichia coli* shuttle vectors constructed with *in vitro* mutagenized yeast genes lacking six-base pair restriction sites. *Gene* **1988**, *74*, 527-534.
4. Koch, B.; Mitterer, V.; Niederhauser, J.; Stanborough, T.; Murat, G.; Rechberger, G.; Bergler, H.; Kressler, D.; Pertschy, B. Yar1 protects the ribosomal protein Rps3 from aggregation. *J. Biol. Chem.* **2012**, *287*, 21806-21815.
5. Ishida, T.; Kinoshita, K. PrDOS: prediction of disordered protein regions from amino acid sequence. *Nucleic Acids Res.* **2007**, *35*, W460-464.
6. Linding, R.; Jensen, L.J.; Diella, F.; Bork, P.; Gibson, T.J.; Russell, R.B. Protein disorder prediction: implications for structural proteomics. *Structure* **2003**, *11*, 1453-1459.
7. Jumper, J.; Evans, R.; Pritzel, A.; Green, T.; Figurnov, M.; Ronneberger, O.; Tunyasuvunakool, K.; Bates, R.; Zidek, A.; Potapenko, A., et al. Highly accurate protein structure prediction with AlphaFold. *Nature* **2021**, *596*, 583-589.
8. Varadi, M.; Anyango, S.; Deshpande, M.; Nair, S.; Natassia, C.; Yordanova, G.; Yuan, D.; Stroe, O.; Wood, G.; Laydon, A., et al. AlphaFold Protein Structure Database: massively expanding the structural coverage of protein-sequence space with high-accuracy models. *Nucleic Acids Res.* **2022**, *50*, D439-D444.
9. Pettersen, E.F.; Goddard, T.D.; Huang, C.C.; Couch, G.S.; Greenblatt, D.M.; Meng, E.C.; Ferrin, T.E. UCSF Chimera-a visualization system for exploratory research and analysis. *J. Comput. Chem.* **2004**, *25*, 1605-1612.

Table S1. Yeast strains used in this work

Strain	Relevant genotype	Source
W303-1B	*MATα ade2-1 his3-11,15 leu2-3, 112 trp1-1 ura3-1*	[1]
JuCY1[a]	As W303-1B but *dbp7*::kanMX4	This work
YKL500	As W303-1B but NOP58-yEmCherry::natNT2 *ade3*::kanMX4	[2]

[a] This strain was transformed with different plasmid-borne *DBP7* alleles to study growth and ribosome biogenesis.

Table S2. Plasmids used in this work

Name	Relevant information	Source
YCplac22	*CEN, TRP1*	[3]
YCplac22-HA-DBP7	HA-*DBP7, CEN, TRP1*	This work
YCplac22-HA-dbp7ΔN10	HA-*dbp7ΔN10, CEN, TRP1*	This work
YCplac22-HA-dbp7ΔN162	HA-*dbp7ΔN162, CEN, TRP1*	This work
YCplac22-HA-dbp7N693	HA-*dbp7ΔC694-742, CEN, TRP1*	This work
YCplac22-HA-dbp7N635	HA-*dbp7ΔC636-742, CEN, TRP1*	This work
YCplac22-HA-dbp7ΔNLS	HA-*dbp7ΔNLS, CEN, TRP1*	This work
pADH111-$(GA)_5$-3xyEGFP	C-terminal yEGFP tag, *CEN, LEU2*	[4]
pADH111-SV40NLS-$(GA)_5$-3xyEGFP	SV40NLS-yEGFP, *CEN, LEU2*	This work
pADH111-DBP7.N162-$(GA)_5$-3xyEGFP	DBP7.N162-$(GA)_5$-3xyEGFP, *CEN, LEU2*	This work
pADH111-DBP7.N162(ΔNLS)-$(GA)_5$-3xyEGFP	DBP7.N162(ΔNLS)-$(GA)_5$-3xyEGFP, *CEN, LEU2*	This work
YCplac111-DBP7-$(GA)_5$-3xyEGFP	DBP7-$(GA)_5$-3xyEGFP, *CEN, LEU2*	This work
YCplac111-DBP7ΔNLS-$(GA)_5$-3xyEGFP	DBP7ΔNLS-$(GA)_5$-3xyEGFP, *CEN, LEU2*	This work
YCplac111-DBP7ΔN162-$(GA)_5$-3xyEGFP	DBP7ΔN162-$(GA)_5$-3xyEGFP, *CEN, LEU2*	This work

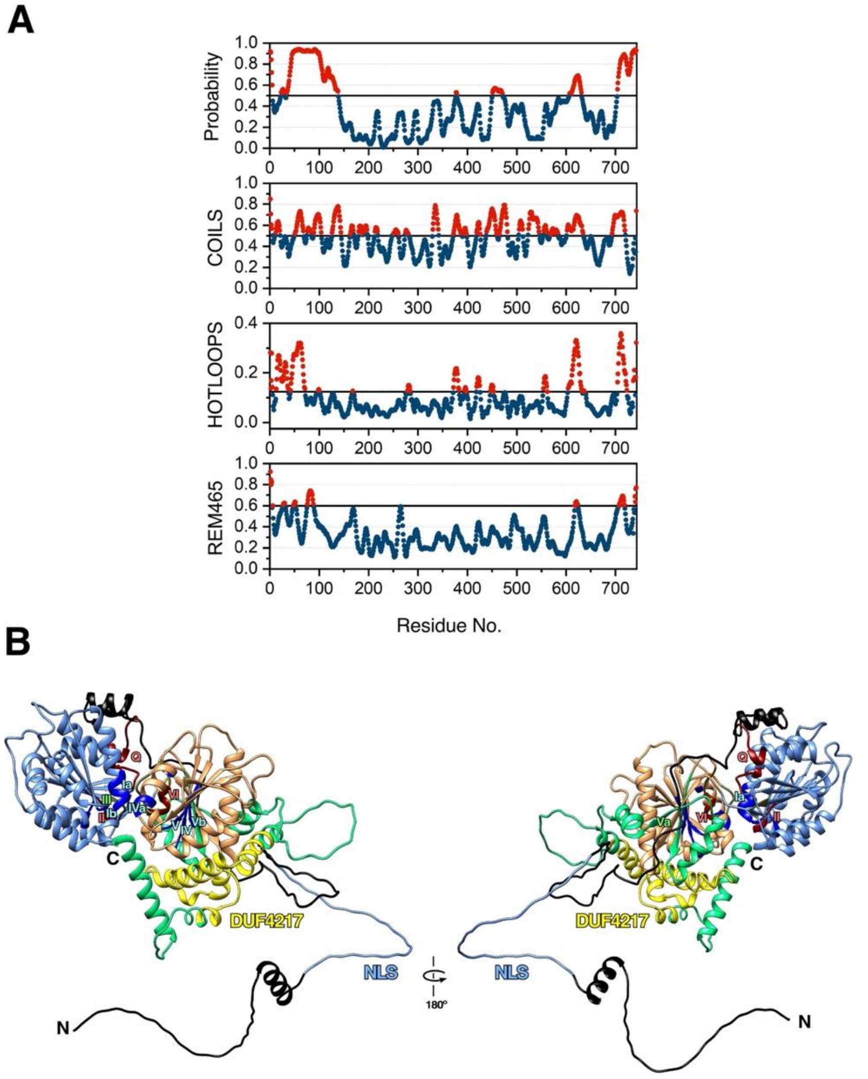

Figure S1. Prediction of intrinsically disordered regions in Dbp7. (**A**) Prediction of the presence of disordered regions. Horizontal lines indicate disorder thresholds in each prediction engine. Red and blue dots indicate residues above and below the disorder threshold, respectively. Predictions using PrDOS (top panel) [5] and DisEMBL 1.5 (second, third and bottom panels showing respectively disorder predictions using the loops/coils, hot-loops or Remark-465 definitions, respectively) [6] are shown. (**B**) Structure prediction of Dbp7 using the PDB file provided for the Dbp7 protein of *Saccharomyces cerevisiae* (strain S288c) by Alpha Fold Protein Structure Database [7,8]. The cartoon was generated with the UCSF Chimera program [9]. The RecA-1 and RecA-2 domains from the helicase core are coloured in blue and gold, respectively. Different motifs are indicated and highlighted in red, blue and green. The N-terminal extension is coloured in black, except the NLS that is coloured in pale blue. The C-terminal extension is coloured in green, except the DUF4217 domain that is coloured in yellow.

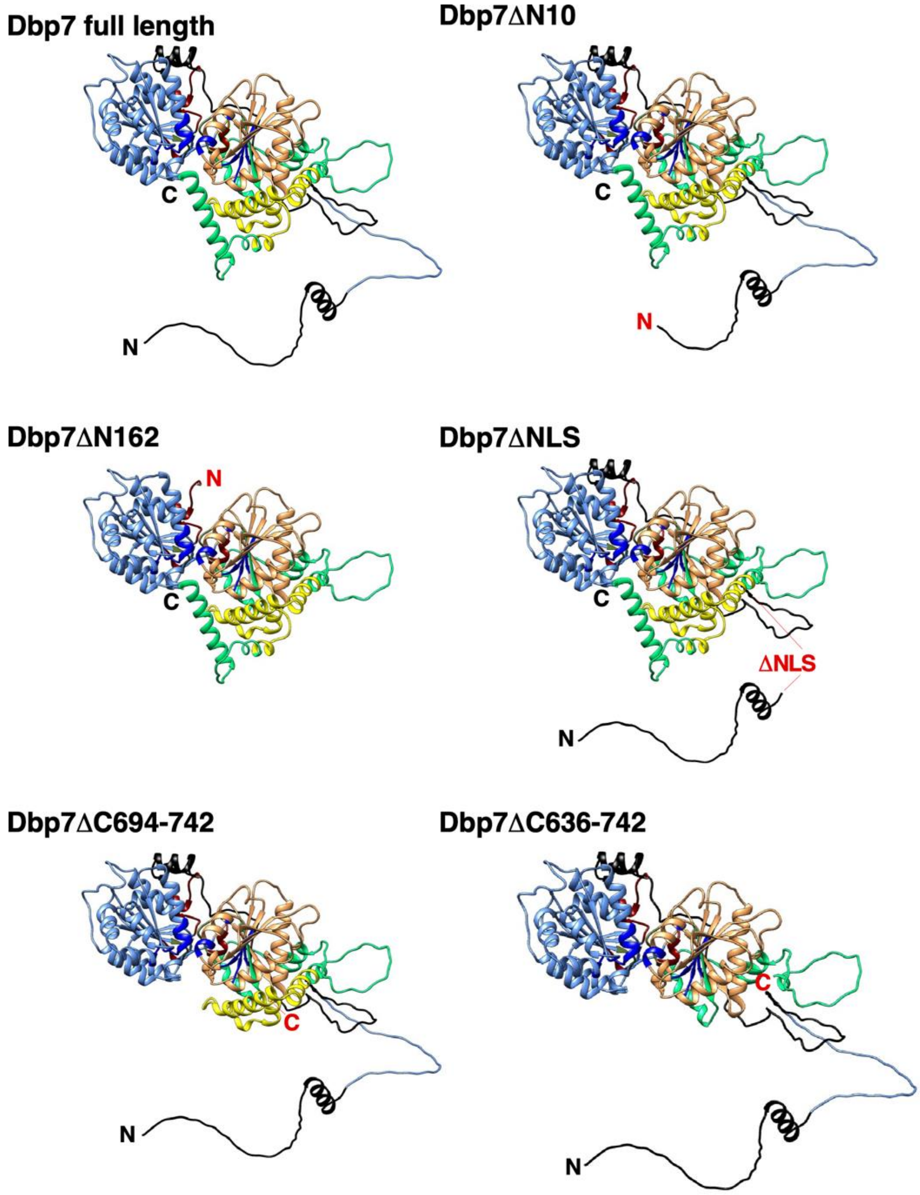

Figure S2. Cartoon representation of the structure of the different truncated versions of Dbp7 proteins of this study. Note that the implicated residues were hidden from the predicted structure of the full-length wild-type Dbp7 protein deposited in Alpha Fold Protein Structure Database. No simulation of how the truncations modified the structure of the remaining protein sequence was undertaken.

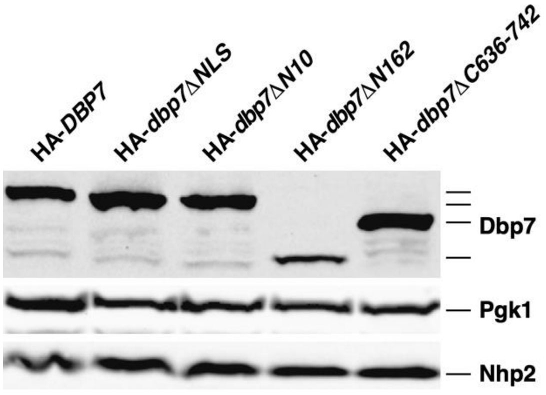

Figure S3. Steady-state levels of the different truncated Dbp7 proteins. Strain JuCY1 transformed with different plasmid-borne *DBP7* alleles: HA-*DBP7* (wild-type control), HA-*dbp7ΔNLS,* HA-*dbp7ΔN10*, HA-*dbp7ΔN162*, and HA-*dbp7ΔC636-742* was grown in liquid SD-Trp medium at 30 °C and harvested at an OD_{600} of 0.8; whole cell extracts were prepared and equivalent amounts of protein from the different cell extracts were subjected to western blotting analyses with antibodies against the HA epitope. Pgk1 and Nhp2 were detected using specific antibodies and used as loading controls.

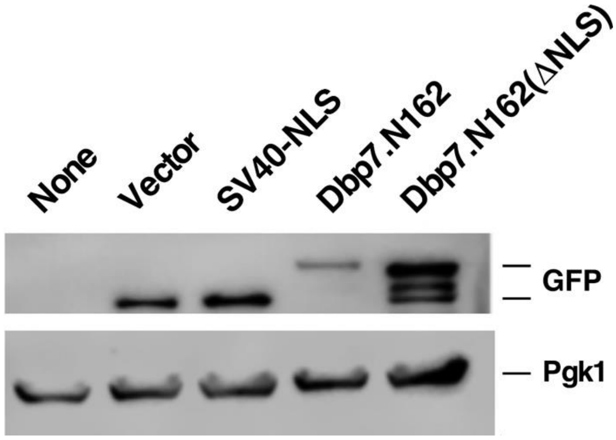

Figure S4. Immunodetection of GFP-fused N-terminal Dbp7 constructs and the respective positive and negative controls. Whole cell extracts were prepared from YKL500 cells transformed with the indicated constructs: None (untransformed cells), empty pADH111-$(GA)_5$-3xyEGFP plasmid (Vector), pADH111-derived plasmid containing the NLS of the SV40 large T-antigen fused to 3xyEGFP (SV40-NLS), pADH111-derived plasmid containing the N-terminal domain of Dbp7 (from M1 to M162) fused to 3xyEGFP (Dbp7.N162) and pADH111-derived plasmid containing the N-terminal domain of Dbp7 (from M1 to M162) but lacking the segment from V48 to S78 (Dbp7.N162(ΔNLS)). Transformants were grown to exponential phase in liquid SD-Leu medium at 30 °C and whole cell extracts were prepared. Equal amounts of extracts were resolved by SDS-PAGE and analysed by western blotting using a specific anti-GFP antibody. Pgk1, which was revealed with a monoclonal anti-Pgk1 antibody, was used as a loading control.

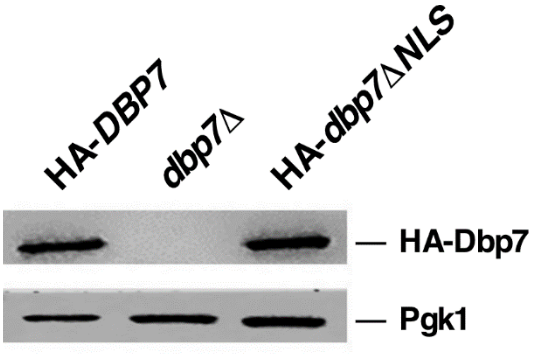

Figure S5. Detection of the HA-Dbp7ΔNLS protein variant lacking the V48 to S78 sequence. Whole cell extracts were prepared from the indicated strains, which were grown to exponential phase in liquid SD-Trp medium at 30 °C. Equivalent amounts of extracts were analysed by western blotting using a specific anti-HA antibody. As a loading control, Pgk1, which was revealed with a monoclonal anti-Pgk1 antibody, was used.

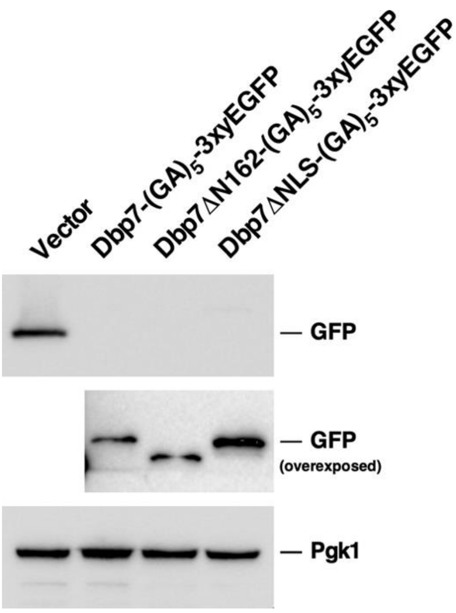

Figure S6. Detection of the different GFP-tagged Dbp7 variant proteins. Whole cell extracts were prepared from the YKL500 strain expressing the indicated, plasmid-borne GFP-tagged Dbp7 constructs under control of the cognate *DBP7* promoter. In addition, YKL500 cells were also transformed with the pADH111-(GA)$_5$-3xyGFP vector, which expresses a triple GFP from the strong *ADH1* promoter. Cells were grown to exponential phase in liquid SD-Leu medium at 30 °C. Equivalent amounts of extracts were analysed by western blotting using a specific anti-GFP antibody. Pgk1, which was revealed with a monoclonal anti-Pgk1 antibody was used as a loading control. Note the difference of the expression of the (GA)$_5$-3xyEGFP reporter from either the *ADH1* or the *DBP7* promoter. The GFP blot was overexposed (ca. 100-fold) to visualize the levels HA-*dbp7ΔC694-742*, HA-*dbp7ΔC636-742*, HA-*dbp7ΔC694-742*, HA-*dbp7ΔC636-742*,of the GFP-tagged Dbp7 variant proteins.

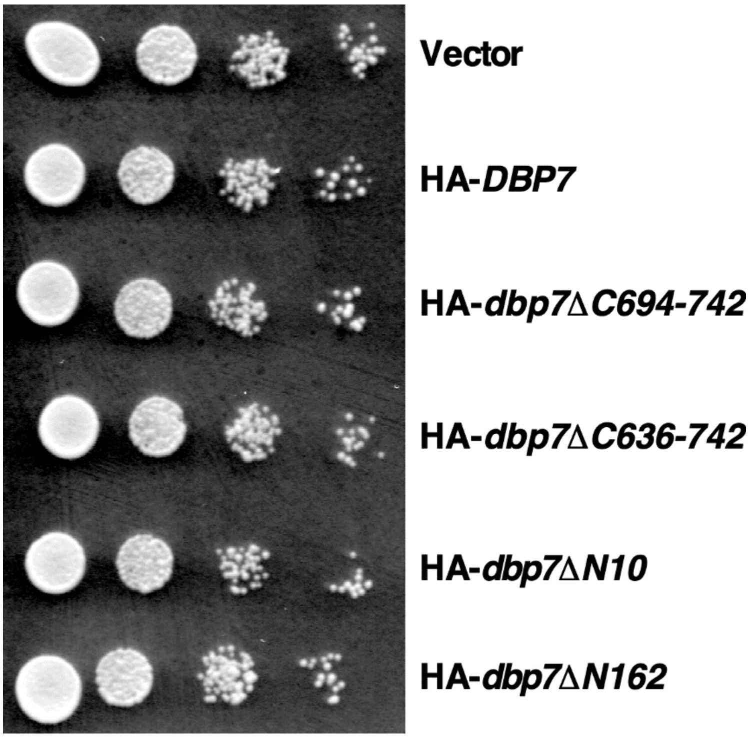

Figure S7. The C-terminal truncations of Dbp7 are not dominant negative over wild-type Dbp7. Growth test of a wild-type W303-1B strain transformed with an empty YCplac22 vector (Vector) or different plasmids expressing the following *DBP7* alleles: HA-*DBP7* (wild-type control), HA-*dbp7ΔC694-742*, HA-*dbp7ΔC636-742*, HA-*dbp7ΔN10* and HA-*dbp7ΔN162*. Strains were serially diluted fivefold and spotted on SD-Trp plates, which were incubated at 30 °C for 2.5 days.
